# Effects of Cu/Zn Superoxide Dismutase (*sod1*) Genotype and Genetic Background on Growth, Reproduction and Defense in *Biomphalaria glabrata*

**DOI:** 10.1371/journal.pntd.0001701

**Published:** 2012-06-19

**Authors:** Kaitlin M. Bonner, Christopher J. Bayne, Maureen K. Larson, Michael S. Blouin

**Affiliations:** Department of Zoology, Oregon State University, Corvallis, Oregon, United States of America; Biomedical Research Institute, United States of America

## Abstract

Resistance of the snail *Biomphalaria glabrata* to the trematode *Schistosoma mansoni* is correlated with allelic variation at copper-zinc superoxide dismutase (*sod1*). We tested whether there is a fitness cost associated with carrying the most resistant allele in three outbred laboratory populations of snails. These three populations were derived from the same base population, but differed in average resistance. Under controlled laboratory conditions we found no cost of carrying the most resistant allele in terms of fecundity, and a possible advantage in terms of growth and mortality. These results suggest that it might be possible to drive resistant alleles of *sod1* into natural populations of the snail vector for the purpose of controlling transmission of *S. mansoni*. However, we did observe a strong effect of genetic background on the association between *sod1* genotype and resistance. *sod1* genotype explained substantial variance in resistance among individuals in the most resistant genetic background, but had little effect in the least resistant genetic background. Thus, epistatic interactions with other loci may be as important a consideration as costs of resistance in the use of *sod1* for vector manipulation.

## Author Summary

Driving resistance genes into vector populations remains a promising but underused method for reducing transmission of vector-borne diseases. Understanding the genetic mechanisms governing resistance and how resistance is maintained in vector populations is essential for the development of resistant vectors as a means of eradicating vector-borne diseases. We investigated the utility of one gene (cytosolic copper-zinc superoxide dismutase - *sod1*) for driving resistance associated alleles into populations of the snail *Biomphalaria glabrata*, a vector of the trematode parasite of humans, *Schistosoma mansoni*. Under controlled laboratory conditions we found no evidence for costs of resistance associated with carrying the most resistant allele at *sod1* (in terms of growth, fecundity, or mortality). However, we did find a strong effect of genetic background on how strongly *sod1* genotype influences resistance. Thus, epistatic interactions with other loci may be as important a consideration as costs of resistance in the use of *sod1* for vector manipulation in the field.

## Introduction

Although vector-borne diseases account for approximately one-sixth of the global human disease burden [1], [2], we still lack effective drugs and vaccines for many of these diseases. Even when effective drugs are available, high-risk populations often cannot be adequately treated due to a lack of funding and infrastructure in the heavily impacted countries [1], [3]. Therefore, in the absence of vaccines, eradication efforts that include both drug therapy and vector control can be the most effective approach [4]. Vector control methods most often utilize chemicals for eradication [1], [4]. This approach has obvious drawbacks because it results in habitat degradation and risk of human exposure to pesticides. Also, recurrent pesticide application is often necessary because it is nearly impossible, with a single treatment, to completely remove all possible vector individuals from an epidemiologically relevant site [5].

Recent advances in understanding the genetics of host-parasite interactions have led to increased interest in driving resistance genes into susceptible vector populations [6]–[11]. In this context, the term “resistance” describes a continuously varying trait we define as the probability of becoming infected after being challenged by a parasite, rather than to mean the absolute inability to become infected (i.e. a population or genotype can have high or low average resistance). Making vector populations more resistant to infection could be a better long-term solution and an ecologically safer way of breaking transmission cycles. Unfortunately, this approach faces major population-genetic hurdles. A non-exhaustive list includes: (1) genotype-by-environment (GxE), where the performance of a gene or gene(s) of interest depends on environmental conditions such that interactions can affect how a resistance gene performs in the field versus in the lab [12]–[16], (2) parasites and hosts are genetically more variable in the field, and there can be interactions between host genotypes and parasite genotypes (genotype-by-genotype (GxG) interactions; [16]–[19]), (3) genetic background can influence how a resistance gene performs in a natural versus a lab population. In other words, the gene of interest may perform differently depending on the genomic context in which it is interacting (epistasis), and (4), there may be a cost of resistance such that natural selection in the absence of parasites favors the “wild-type” alleles that we wish to replace.

Cost of resistance may be a particularly vexing problem for resistance-gene introduction programs. Such costs have been demonstrated in many host-parasite systems (reviewed in [20]–[26]). Nevertheless, costs of resistance are not universal [8], [27]–[31], and they may be context dependent (e.g. revealed only in stressful environments; [12], [32]–[36]). Costs of resistance presumably involve a reallocation of metabolic resources between one or more of the following life-history components: reproduction, growth, and somatic maintenance/immune function [24], [26], [37], [38]. Also, the severity of the cost should depend on the particular mechanism of resistance [29], [39]. For example, it was predicted that mechanisms involving over-expression of particular genes might be among the most costly [39].

This study was designed to measure costs of resistance and epistatic effects of genetic background associated with a single locus in *Biomphalaria glabrata*, a snail vector of the human pathogen *Schistosoma mansoni*. Schistosomiasis is responsible for approximately 200,000 deaths yearly, with 200 million people infected worldwide [40]–[42]. *B. glabrata* is a facultative, hermaphroditic freshwater pulmonate snail that occurs throughout much of the New World tropics [43]–[45]. The *B. glabrata/S. mansoni* system is a well-established model for investigating host-parasite interactions in a controlled laboratory setting [46].

Resistance to *S. mansoni* infection in *B. glabrata* is highly heritable in many lab and field populations, and is almost certainly controlled by multiple loci [47]–[52]. The expression patterns of known immune-related genes have been found to differ between individuals from more resistant and less resistant strains when each is challenged with the same strain of parasite [53]–[59]. However, to date only a single locus has been identified at which allelic variation clearly associates with resistance to the parasite: copper-zinc superoxide dismutase (*sod1*) [60], [61]. SOD1 is a ubiquitous protein involved in several cellular functions including signaling and immune response [62]–[65]. Among the various functions of SOD1, it catalyzes the reduction of highly reactive superoxide (O_2_^−^) to hydrogen peroxide (H_2_O_2_). Hydrogen peroxide is a known cytotoxic component of the oxidative burst, which is the primary defense mechanism for parasite clearance in molluscs [46], [66], [67]. When a schistosome invades a snail, hemocytes surround the invading parasite and are thought to generate H_2_O_2_ as part of the killing mechanism [46], [66], [68]. Consistent with this hypothesis, increased H_2_O_2_ production was correlated with the difference in resistance between snails from the M-line strain and the more resistant 13–16-R1 strain [46], [68]. An *sod1* allele present in the 13–16-R1 strain was over-expressed relative to the other alleles, and correlated with a more effective defense against parasite infection [46], [61], [69]. More recently, Moné et al. [70] demonstrated a correlation between the ability of certain strains of *B. glabrata* to produce reactive oxygen species and the anti-oxidant defenses of their respective compatible *S. mansoni* strains. Thus, loci involved in the oxidative burst, such as *sod1*, may be very important in the evolution of schistosome-snail interactions. Therefore, *sod1* is a promising candidate locus for driving resistance alleles into susceptible natural populations of snails.

Although *sod1* seems a favorable candidate for genetic manipulation of snail populations, there are two reasons why one might expect a cost of resistance associated with the allelic polymorphism at *sod1*. First, increased expression of any gene is likely to be costly [39]. Second, increased expression of *sod1* might incur a cost due to increased oxidative stress on the host [71], [72]. Therefore, investigating the fitness costs associated with allelic variation at *sod1* is an important first step in evaluating the potential use of *sod1* for creating highly resistant vector populations in the field.

## Methods

### Study population

We used a population of the 13–16-R1 strain of *B. glabrata* that has been maintained as a large population (hundreds) in C.J. Bayne's lab at Oregon State University since the mid-1970s. The 13–16-R1 strain was reportedly created by crossing highly resistant strains of snails isolated from Brazil and Puerto Rico [47] but it has been in culture for so long in so many laboratories that its history is not entirely clear. Our population has been maintained in the absence of parasite exposure, and therefore under relaxed selective pressure in regards to resistance to *S. mansoni*. *B. glabrata* is a facultative self-fertilizing hermaphrodite such that snails will preferentially outcross when given access to a mate, but when isolated will usually reproduce through self-fertilization (e.g. [73]–[75]; our laboratory population is in Hardy-Weinberg Equilibrium for *sod1* and microsatellite loci: [61], [69]; unpub. data). We recently created 52 inbred lines: we started with haphazardly picked juvenile snails and completed three generations of selfing using a single offspring from each self-fertilization event to begin the next generation. The inbred lines are mostly fixed for one of three alleles of *sod1 A*, *B* and *C*, as described in [61]. These lines also vary substantially for resistance within each *sod1* genotypic class (*AA*, *BB*, and *CC*). That there are highly resistant and highly susceptible lines within each *sod1* class suggests that other loci besides *sod1* have a large effect in determining resistance. These inbred lines can be used to compare directly the fitness effects of carrying a specific genotype at *sod1* and the effects of genetic background on the association between resistance and *sod1* genotype.

### Breeding scheme

**Figure 1:**
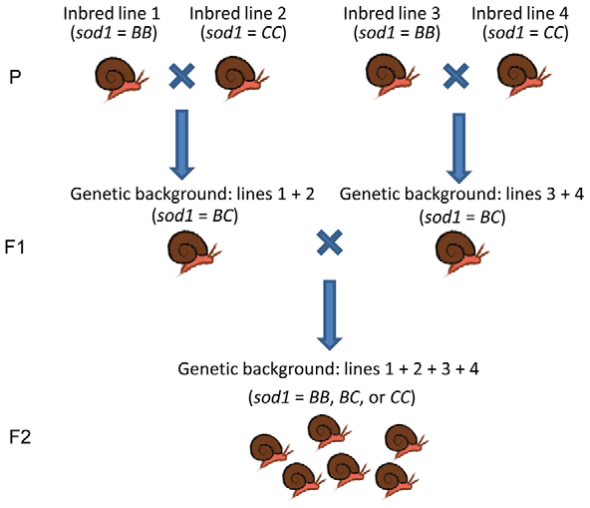
Breeding scheme for generating F2 populations with different genetic backgrounds. We created F2 populations by crossing inbred lines (3 generations of selfing) that were fixed for *BB* or *CC* genotypes. F1 offspring from unique inbred line crosses (fixed for the *BC* genotype) were then bred to generate outbred F2 populations that were segregating for *BB*, *BC*, *CC* genotypes in the expected Mendelian ratio. This was done three times to generate three different genetic backgrounds that differ in average resistance.

**Figure 2:**
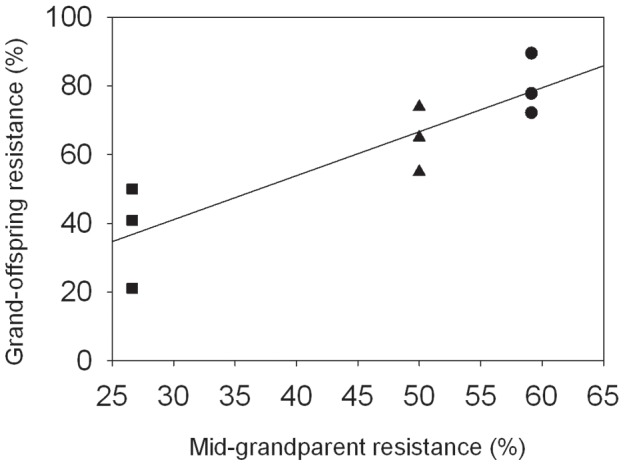
Resistance of genetic background as a function of average resistance of grandparental inbred lines. Mid-grandparent resistance was estimated by averaging the resistance of the four inbred, grandparental lines (determined previously). The resistance of each genetic background (Grand-offspring resistance) was estimated by parasite challenges done in triplicate (n = 24×3) for each background (• genetic background 1, ▴ genetic background 2, and ▪ genetic background 3).

Several inbred lines were used to create three outbred F2 populations, each of which was segregating for the *B* and *C* allele (Figure 1). We hereafter refer to these three F2 populations as “genetic backgrounds” because we wanted to know if the phenotypic effects of variation at *sod1* depend on the genomic context in which those alleles are expressed. These F2 individuals were then used to evaluate the effects of *sod1* allele on life history traits and resistance. Inbred lines were chosen so that the three populations differed in average resistance. *BB* and *CC* fixed lines were chosen because the B allele confers the highest resistance and the *C* allele the lowest [61]. Additionally, in hemocytes (the defense cells) the *B* allele is constitutively over-expressed relative to the other two alleles [69]. To create the three F2 populations, we paired an individual from an inbred line fixed for the *B* allele with an individual from an inbred line fixed for the *C* allele (*BB*×*CC*), which resulted in offspring that were heterozygous at *sod1* (*BC*). Three unique *BB* and *CC* inbred lines were used, and each cross was completed in triplicate with unique individuals (n = 9 crosses). To compare directly the effects of carrying the *BB* and *CC* genotypes within a family and among different backgrounds, we paired heterozygous offspring from each initial cross with a heterozygous individual from a different initial cross using a factorial design. This resulted in three different F2 populations of outbred individuals that had the same *sod1* genotypes, but in different genetic backgrounds (Figure 1). The F2 individuals in each of the three populations carried the *BB*, *BC* and *CC* genotypes in the expected (1∶2∶1) Mendelian ratios (*sod1* genotypes were verified by sequencing). We used these F2 individuals to test the effects of *sod1* genotype on fecundity, growth and resistance in each of the three genetic backgrounds. Our three populations (genetic backgrounds) differed in overall resistance (77.8%, 63.8%, and 38.9%), which strongly correlated with the resistance of their grandparents (the original inbred lines) (Figure 2).

### Resistance

For each F2 population (genetic background), a total of 72 individuals were haphazardly chosen from a pool of offspring from the final set of crosses. We exposed single juvenile snails (4–5 mm diameter) to five *S. mansoni* strain PR-1 miracidia in 3 mL of artificial spring water (ASW; [76]) for two hours at 26°C, in 12-well culture plates. The PR-1 strain has been maintained in Syrian hamsters and the M-line (Oregon) strain of *B. glabrata* snails by the Bayne lab for 36 years. Challenged individuals were then reared in moderately dark tubs in groups of 24, with three replicate tubs for each background (n = 72). We examined the snails for infection at six, nine, and eleven weeks (we rarely see shedding after 11 weeks). Each examination week we induced cercarial shedding (parasite emergence) by exposing snails individually in 3 mL of ASW to direct fluorescent light for two hours at 26°C in 12-well culture plates. The presence of cercarial shedding indicated a positive infection. Infected snails were preserved in 95% ethanol (EtOH), and non-infected snails were returned to rearing tubs after each assay. After the final cercarial shedding attempt (eleven weeks) we preserved the remaining snails, and all tissue samples were processed for *sod1* genotyping (described below in ‘Molecular Methods’ section). Resistance to parasite infection was scored in each tub group as the percentage of snails that did not shed cercariae by eleven weeks post-challenge. Snails that died prior to shedding assays were excluded from the experiment. Average mortality observed from the parasite challenge ranged from 8–12% among tubs, and did not differ among genetic backgrounds (One-way ANOVA, p = 0.442).

### Growth

We collected single egg masses (n = 58) from Styrofoam substrate within 48-hours of egg mass deposition from individual pairs of the final set of crosses (i.e. embryos in the eggs are F2s). The single egg masses were reared individually and allowed to hatch. We measured offspring size (diameter of the shell) twelve weeks after egg mass deposition. All snails were then preserved in 95% ethanol for subsequent *sod1* genotyping. Clutch sizes (the numbers of eggs/embryos in single egg masses) ranged from 2 to 34 (n = 58). Initial analysis revealed that average offspring size was correlated with clutch size, (adjusted R^2^ = 0.363, *P*<0.001) suggesting a strong density-dependent effect of number of snails per bowl on growth (same effect across all genetic backgrounds). Therefore, we restricted our analysis of effects of *sod1* genotype to the offspring of clutch sizes between 13–17 eggs/embryos (there was no association between clutch size and snail size within that limited range of clutch sizes; adjusted R^2^ 0.001, *P* = 0.28). We compared snail growth from 3–4 clutches in each genetic background (background 1: n = 45, background 2: n = 57, background 3: n = 58).

We also measured growth (shell diameter) in snails that were raised individually for 32 weeks as part of the egg production and hatch success experiments described below (hereafter referred to as “late growth” compared to the “early growth” measures described in the above experiment).

### Fecundity

As in the growth study, we collected egg masses from individual pairs of the final crosses (i.e. the F2 offspring). From each population, we haphazardly chose 50 sexually immature offspring (4–5 mm shell diameter). Each snail was reared singly and a portion of a tentacle was excised to determine its *sod1* genotype. We then randomly chose ten juveniles of each genotype (*BB*, *BC*, and *CC*) from each set of 50 genotyped snails, and reared them individually for subsequent fecundity comparisons (i.e. n = 30 per genetic background). Because *B. glabrata* is a facultative self-fertilizing hermaphrodite, we provided a mate to each snail prior to measuring egg production and hatch success to ensure offspring were not the result of selfing (because inbreeding depression is expected to affect egg survival). We chose to mate the genotyped individuals with snails from an isogenic inbred population to keep consistent the relative contribution of the “male-acting” snail to egg production. The isogenic inbred individuals were from a population of inbred M-line strain of *B. glabrata* established at the University of New Mexico through 32 generations of selfing (Si-Ming Zhang *pers comm.*). Because the M-line and F2 offspring look morphologically similar, we marked the M-line snails with a white dot using nail polish 24 hours prior to mating.

All snails were individually reared until reproductively active, as determined by the presence of well-formed egg masses containing developing embryos. *B. glabrata* preferentially use allosperm for fertilization and store sperm for up to 10 days [74]. Consequently, each snail was paired with a size-matched, painted, inbred M-line individual for one week, then separated and allowed to lay eggs for one week in a new cup. These eggs were thus presumably fertilized by allosperm, even though layed in the absence of a partner [73]–[75]. Egg numbers were counted at the end of each 1-week laying period, after which snails were re-paired with a different mate. We continued the mating/laying schedule for ten weeks, resulting in five one-week accumulated egg production measurements from each snail. We present the sum of the five one-week egg accumulation measures as the total egg production for each snail over five weeks.

### Hatch success

We examined egg hatch success in the same set of genotyped individuals in which we surveyed egg production. Each snail was paired with a size-matched painted inbred M-line individual for 48 hours, and then isolated in a new cup. Two egg masses from each snail were carefully collected 72 hours post-transfer and reared individually (n = 180). Egg masses were surveyed for total egg count upon collection, and final hatch counts were conducted six weeks later. Hatch success (percent of eggs hatched at six weeks) from the two egg masses was averaged for each snail.

### Mortality

In addition to measuring egg production and egg hatch, we also monitored mortality at eight and twelve months in the same set of F2 snails used for the egg production and hatch success experiments. Mortality was measured as percent of individuals from each *sod1* genotype alive at the time of census for each genetic background.

### Snail rearing conditions

All snails were reared in an environmentally controlled room kept at 26°C and on a 12 hr day/12 hr night light cycle with full spectrum light. Snails were fed green leaf lettuce *ad libitum* throughout all experiments. In experiments other than those in which we measured resistance, egg masses and snails were reared, mated, and maintained in 500 mL cups with 300 mL of ASW. Complete water changes were carried out weekly. When generating the three different populations (i.e. the three different genetic backgrounds) for the fecundity experiments, the egg masses (and offspring) were reared in 2 L of ASW in aerated, lidded 1-gallon, clear plastic boxes (IRIS, USA). The egg masses monitored in the hatch success experiment were reared in petri-dishes (100×15 mm) with 5 mL of ASW. Finally, in the resistance assay we reared exposed snails in moderately dark, lidded 3-gallon plastic tubs (Dark Indigo Rubbermaid Roughneck boxes). Each contained 7.5 L of aerated dechlorinated water supplemented with 10 mL of calcium carbonate shell hardening solution (30 mg Ca^++^/L). Half of the water was changed with dechlorinated water between each infection assay.

### Molecular methods

We extracted genomic DNA from snail head foot tissue following the CTAB protocol [77], and used chelex extraction methods for tentacle tissue. *sod1* genotype was determined using fragment analysis on an ABI 3730 capillary sequencer following amplification with AmpliTaq (Applied Biosystems, Inc.) (F-(VIC) - TCA TTG GTC GCA GCT TAG TG, R - GTC CTG TCA TGT AGC CAC CA). The *B* and *C* alleles are differentiated by a two base-pair (bp) insertion/deletion in the fourth intron that is fully resolved by the capillary system (the full sequences for the fourth intron are available for the *B* and *C* allele on NCBI GenBank from [61]). Sequence analysis of a subset of samples corroborated fragment analysis methods. Fragment analysis peaks were visualized using GENOTYPER (Applied Biosystems, Inc.), and sequence data were analyzed using SEQUENCHER (GeneCodes, Inc.).

### Statistical methods

Data were assessed for normality (Shapiro-Wilk) and equal variance. To examine the effects of genetic background on the association between carrying the B allele and resistance to parasite infection we used generalized linear models (logit function) to compare resistance (coded as a binomial response for each snail, infected = 1, not infected = 0) among genetic backgrounds and *sod1* genotypes. We used regression coefficients from individual logistic regressions to quantify the relative effect sizes of substituting one allele for another in each of the genetic backgrounds. We compared fitness measures (growth rate, egg production, and hatch success) among genetic backgrounds and genotypes using two-way ANOVAs and Tukey post-hoc tests. For mortality we used generalized linear models (logit function, surviving snail at time of census = 1, dead snail = 0). No transformations were needed to normalize any of these data. We defined significance at the level of alpha = 0.05. For data analyses, we used the statistical packages SPlus version 8.1 for Windows (TIBCO Software, Inc) and SigmaPlot for Windows version 11.0 (Systat Software, Inc).

## Results

### Resistance

**Figure 3:**
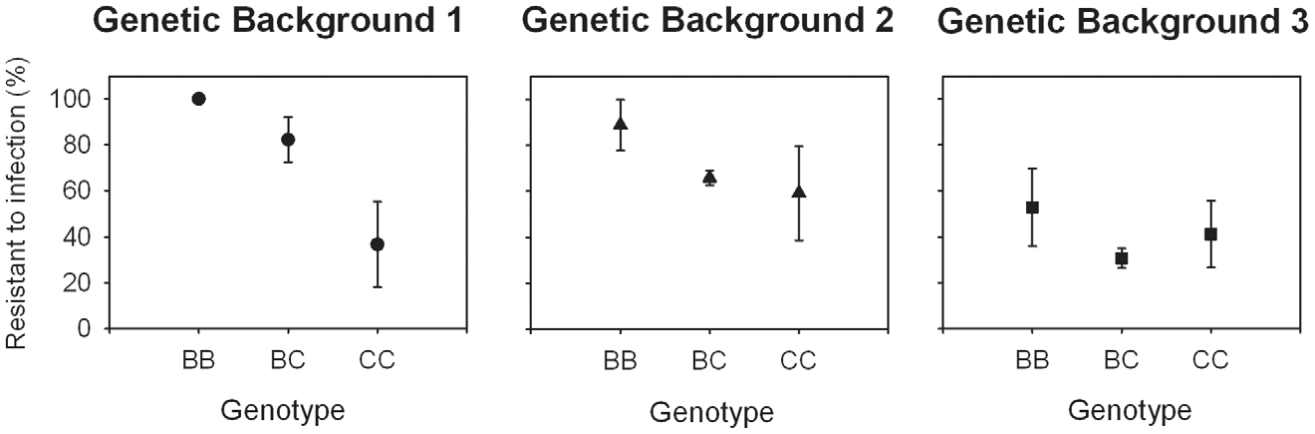
Effects of genetic background and *sod1* genotype on resistance to infection. Graphs illustrate the average resistance of each genotypic class within each background after a challenge with five PR-1 *S. mansoni* miracidia. Resistance means are the averages of three replicates (tubs starting with n = 24 snails each). Error bars represent 1±SE (background 1: n = 55 (*BB* = 10, *BC* = 36, *CC* = 9), background 2: n = 63 (*BB* = 8, *BC* = 39, *CC* = 16), background 3: n = 63 (*BB* = 14, *BC* = 35, *CC* = 14)). There were significant main effects of genetic background, genotype, and interaction between genetic background and *sod1* genotype. (See text for statistical analyses.)

We found main effects of genotype and genetic background, and a background-by-genotype interaction (logit GLM; background: *P* = 0.09, genotype: *P* = 0.003, background×genotype: *P* = 0.022). As expected, the *B* allele was most protective. However, the strength of the association between *sod1* genotype and resistance to infection depended on genetic background. The association was strongest in genetic background 1 and there was a similar but non-significant trend in background 2. In contrast, allelic variation at *sod1* explained little of the variance in resistance in background 3 (Figure 3). Substituting a *B* allele for a *C* allele decreased the odds of infection by 6.2 in genetic background 1, and by 2.5 in genetic background 2 (logit GLM; *P* = 0.0027 and 0.0477, respectively). In genetic background 3 there was no significant additive effect. Thus, the effect of allelic variation at *sod1* on resistance to infection was most important in predicting infection in the genetic background having high average resistance, and was largely irrelevant in the low-resistance genetic background.

### Growth

**Figure 4:**
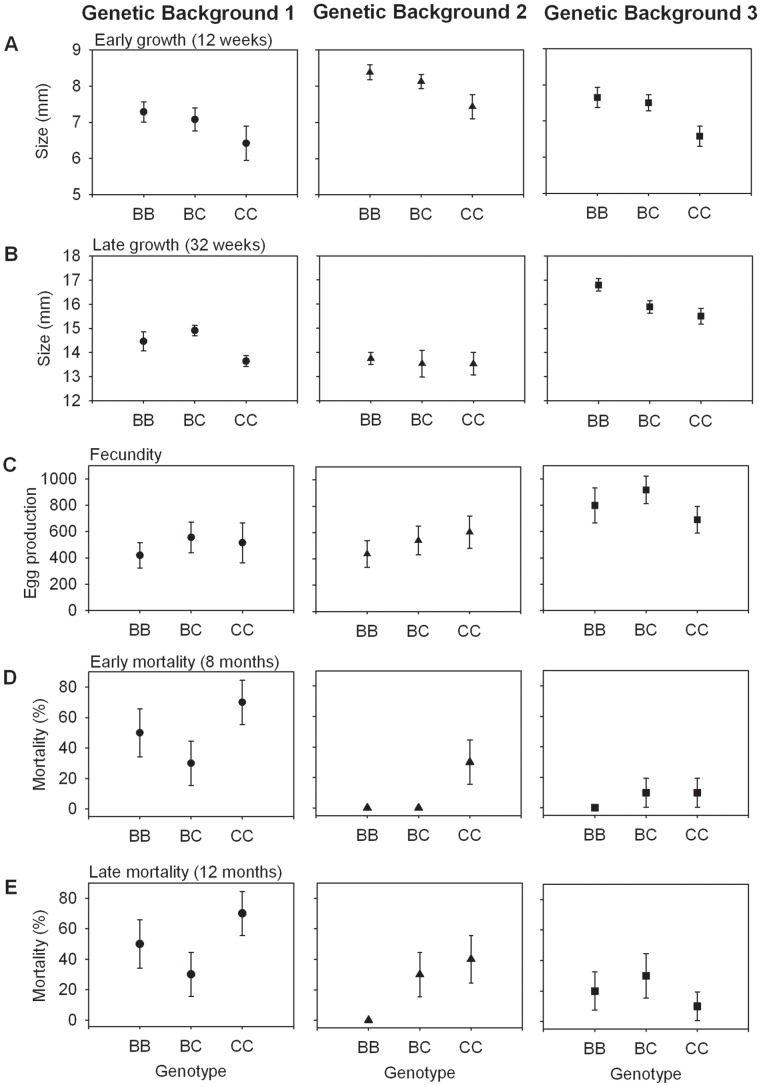
Effect of genetic background and *sod1* genotype on life-history traits. (A) Average size by genotypic class within each lineage at 12 weeks after egg masses were deposited. The points represent the averages of the mean size of individuals of each genotype within each of 3–4 cups (containing 13–17 F2 snails per cup). Error bars represent 1±SE (background 1: n = 45 (*BB* = 16, *BC* = 20, *CC* = 9), background 2: n = 57 (*BB* = 16, *BC* = 28, *CC* = 13) background 3: n = 58 (*BB* = 16, *BC* = 28, *CC* = 14)). Snails with the *CC* genotype grew significantly more slowly than those with *BC* and *BB* genotypes. (B) Average size at 32 weeks of each genotypic class within each lineage. Means are the average of all snails within the genotypic class, and error bars represent 1±SE (background 1: n = 27 (*BB* = 9, *BC* = 10, *CC* = 8), background 2: n = 27 (*BB* = 9, *BC* = 10, *CC* = 8), background 3: n = 30 (*BB* = 10, *BC* = 10, *CC* = 10)). Again, snails with the *CC* genotype grew significantly more slowly than those with *BC* and *BB* genotypes. (C) Average total egg production for five weeks per snail (each raised individually) by genotypic class within each lineage. Means are the average of all snails within the genotypic class, and error bars represent 1±SE (background 1: n = 25 (*BB* = 9, *BC* = 9,*CC* = 7), background 2: n = 27 (*BB* = 9, *BC* = 10, *CC* = 8), background 3: n = 30 (*BB* = 10, *BC* = 10,*CC* = 10)). (D) Mortality at 8-month census of each genotypic class within each lineage. Data points are estimates of the percent mortality in each genotypic class and error bars represent 1±SE on the proportion (for all backgrounds n = 30 (*BB* = 10, *BC* = 10, *CC* = 10)). Snails with the *CC* genotype exhibited significantly greater mortality than those with the *BB* or *BC* genotype. (E) Mortality at 12-month census of each genotypic class within each lineage. Data points are estimates of percent mortality in each genotypic class and error bars represent 1±SE on the proportion (for all backgrounds n = 30 (*BB* = 10, *BC* = 10, *CC* = 10)).

With regard to early growth (size at 12 weeks), we found significant main effects of genetic background and *sod1* genotype, but no interaction effect. Surprisingly, individuals with the *CC* genotype were smaller, on average, than those with *BB* and *BC* genotypes (two-way ANOVA; background: *F_2,151_* = 11.07,*P*<0.001; genotype: *F_2,151_* = 8.11,*P*<0.001; background×genotype: *F_4,151_* = 0.68, *P* = 0.991) (Figure 4A). Thus the *B* allele was associated with faster growth and appeared almost completely dominant to the *C* allele for this trait (Figure 4A).

For late growth (size at 32 weeks), we again found significant main effects of genetic background and genotype, and no interaction (two-way ANOVA; background: *F_2,75_* = 39.8, *P*<0.001; genotype: *F_2,75_* = 3.68, *P* = 0.030; background×genotype: *F_4,75_* = 1.54, *P* = 0.20). The *CC* individuals were still smaller than the *BC* and *BB* individuals, and the *B* allele appeared to act dominantly (Figure 4B).

### Fecundity and hatch success

In regard to egg production, we found a main effect of genetic background, but no main effect of *sod1* genotype and no significant interaction (two-way ANOVA; background: *F_2,73_* = 6.11, *P* = 0.0035; genotype: *F_2,73_* = 0.533, *P* = 0.59; background×genotype: *F_4,73_* = 0.472, *P* = 0.756). The *BB* genotype had the lowest estimated fecundity in genetic backgrounds 1 and 2, but the *CC* genotype had the lowest in background 3 (Figure 4C). However, we examined only 10 individuals per genotype within each genetic background, and thus had low power to detect all but strong main or interaction effects, as evidenced from a post-hoc power analysis. Our calculated effect size for the main effect of genetic background was 0.432, while effect sizes for the main effect of genotype and interaction were only 0.15 and 0.17, respectively. Additionally, our calculated power was 0.95 for the main effect of genetic background but only 0.22 and 0.27 for the main effect of genotype and for the interaction, respectively. Thus, an effect of *sod1* genotype on fecundity would have had to be much stronger than observed to be detected with our sample sizes.

Average hatch success across all genetic backgrounds was 49%, and varied from 35% to 62% among genotypes (Figure S1). We did not find a significant main effect of genetic background or genotype on hatch success (two-way ANOVA; background: *F_2,60_* = 0.47, *P* = 0.62; genotype: *F_2,60_* = 1.52, *P* = 0.23; background×genotype: *F_4,60_* = 0.99, *P* = 0.42). Thus, the *B* allele does not incur an obvious fitness cost associated with egg production (Figure 4C) or offspring hatch success. We note that although our average hatch rate of 49% is on the low side of rates reported in the literature, it is not unusually low (e.g. [78]).

### Mortality

At the 8-month census we found significant main effects of both genetic background and genotype on mortality (logit GLM, background: *P* = 0.002, genotype: *P* = 0.04), but no interaction (drop-in-deviance test, *P* = 0.19). *CC* individuals exhibited greater mortality, averaging 37% across genetic backgrounds, whereas *BB* and *BC* average 17% and 13% respectively (Figure 4D).

At 12 months we again found a significant main effect of genetic background, but the genotype effect was no longer significant (logit GLM, background: *P* = 0.02, genotype: *P* = 0.18), and there was no interaction (drop-in-deviance test, *P* = 0.39). These results suggest there is no cost to having the *B* allele in terms of increased mortality, and a possible advantage in early survival (Figure 4E).

## Discussion

In this study we considered the utility of a resistance-associated locus, cytosolic copper-zinc superoxide dismutase (*sod1*) in *Biomphalaria glabrata*, for vector-mediated control of *Schistosoma mansoni*. We looked for evidence of fitness costs in growth rate and reproduction. We also tested for epistatic effects of genetic background by assessing influence of the *B* and *C* alleles on resistance and on life history traits.

### The effect of *sod1* on resistance depends on other loci in the genome

The association between allelic variation at *sod1* and resistance to infection varied substantially among genetic backgrounds. The three genetic backgrounds differed in average resistance (78%, 64%, and 39%; Figure 2). *sod1* genotype was most predictive in the genetic background having the highest average resistance, and had a negligible effect in the genetic background having the lowest average resistance (Figure 3). Thus, *sod1* appears to interact epistatically with other genes that influence resistance, a result that might help us identify those other loci. That there are other resistance loci segregating in the 13–16-R1 population is evident because inbred lines having identical *sod1* genotypes vary substantially in resistance (Bender and Larson, unpublished observations). Through gene expression studies, several other loci have been identified in *B. glabrata* as being potentially immune relevant [53]–[59], and various physiological differences have been noted between snail strains having high or low resistance to trematode parasites (reviewed in [67]). However, candidates that seem particularly likely to interact with *sod1* as observed here include loci encoding proteins involved in non-self recognition and loci that control other steps in the oxidative burst pathways. Recognition loci are suggested because, as part of the effector mechanism used by the host to attack the parasite, *sod1* would come into play only after the parasite has been recognized. Thus, *sod1* genotype would be irrelevant in a low-recognition background, but very important in a high-recognition background. Possible recognition loci include lectin-like molecules such as FREPs [79]. Loci affecting numbers or some other property of hemocytes might also behave epistatically with *sod1* in a similar manner such that if hemocytes were incompetent (or insufficient in number) to encapsulate the parasite, their ability to produce H_2_O_2_ would be irrelevant.

### No evidence for a cost of resistance at *sod1*

Costs of resistance have been demonstrated in many systems [21]–[26]. Even in *B. glabrata*, there is some evidence that strains with higher resistance to schistosomes differ from strains with lower resistance in components of fitness [49], [50], [80]–[85]. Furthermore, relative to the *A* and *C* alleles, the *B* allele of *sod1* is over-expressed. The SOD1 protein produces H_2_O_2_, a highly reactive species with the potential to damage host tissue as well as the parasite [69]. Thus, it would be no surprise to see a cost of resistance associated with the *B* allele at *sod1*. Nevertheless, here we failed to detect any disadvantage due to the *B* allele in terms of reproduction, and observed an advantage over the *C* allele in terms of growth rate and survival to 8 months post-hatch (Figure 4). Furthermore, there were no significant interactions between *sod1* genotype and genetic background with regard to life history traits. It is also interesting that the *B* allele acted dominantly to the *C* allele for growth rate (Figure 3), a result that might be expected if the difference really results from over-expression of the *B* allele.

Given our data suggest that the *B* allele may confer a slight advantage in terms of growth and early survival, one might wonder why our population has not become fixed for the *B* allele. Possible explanations include: (1) this laboratory maintained population is not in equilibrium and the selection pressure is not strong enough to have driven the allele to higher frequency yet (we have no data on allele frequencies of *sod1* at the founding of this laboratory population); (2) there may be costs to having the *B* allele in other components of fitness that we did not measure; (3) perhaps there are complex interactions among the three major alleles in the population (*A*, *B*, and *C*) that prevent the *B* allele from increasing in frequency (e.g. see p 223–225 in [86]).

### Potential use of *sod1* for vector manipulation: caveats and additional questions

We showed the promising result of no obvious cost, and perhaps a life history trait advantage for the more-resistant allele at *sod1*. Obvious caveats include that our experiments were conducted in a (presumably benign) laboratory setting, and would need to be replicated under field conditions. Other studies have found that costs of resistance are more likely to manifest under specific environmental conditions, such as low food and temperature stress [12], [32], [35], [36]. Of perhaps greater concern is the strong epistatic effect on resistance between *sod1* and other loci in the genome. Defeating an attempted infection is a complex process that involves many steps including recognition, signaling and implementing the effector (killing) mechanisms. SOD1 can participate in both signaling and effector mechanisms, and the products of many loci may need to interact properly to sufficiently clear an infection. Thus, it will be essential to assess the performance of *sod1* in the field and in a variety of other genetic backgrounds.

There are also a number of basic questions, unrelated to those addressed here, about *sod1* and resistance to *S. mansoni* that need to be answered before one could seriously consider using *sod1* for vector manipulation in the field. We still need to prove that the association between resistance and *sod1* alleles is actually causal, and if so, if the protective effect of allele *B* is really owing to its overexpression. It is theoretically possible that *sod1* is not the actual causal locus, but is just in strong linkage disequilibrium with a closely-linked locus that actually controls resistance. This seems unlikely given the association between *sod1* genotype and resistance was discovered using a functional approach (e.g. knocking down H_2_O_2_ production in *B. glabrata* hemocytes increases their susceptibility to infection [66]), but the functional basis of the association still needs to be proven. Additional work to test the causality of the association is underway. In the unlikely event it turns out that another locus is actually causal, then the results of this study are still quite relevant, but for the new locus of interest.

We also do not know yet if the effect of *sod1* we observed is generalizable to other populations/strains of *S. mansoni*. We have only studied the PR-1 strain of *S. mansoni* in interaction with the 13–16-R1 population of *B. glabrata*. It is possible that the protective effect of *sod1* alleles depends on the strain of parasite in addition to the strain of snail. In a similar vein, we also have no data on if, or how, *sod1* genotype affects resistance to other pathogens. A field population of snails interacts with many pathogens in addition to *S. mansoni*, and there could be fitness tradeoffs associated with other pathogens that render the use of *sod1* for vector manipulation ineffective in some environments.

In summary, we have here shown that, in a laboratory setting, there was no obvious cost to having the most protective allele at *sod1*, and perhaps a slight advantage. The generality of this result will need to be verified in other environments, and for other components of fitness. We also demonstrated an effect of genetic background on the association between *sod1* genotype and resistance, a result that points to strong epistatic interactions with other loci in the genome. Clearly *sod1* is not the only locus in the genome that influences resistance. So perhaps vector manipulation will require changes at several interacting loci to insure success. Further work of this sort on *sod1* and other resistance-associated loci will be essential for evaluating the prospects for vector manipulation as a way to control transmission of *S. mansoni*.

## Supporting Information

Figure S1 **Average hatch success of each genotypic class within each lineage.** Means are the average of percent hatch of two clutches per snail across genotypic class, and error bars represent 1±SE (background 1: n = 17 (*BB* = 8, *BC* = 6,*CC* = 3), background 2: n = 24 (*BB* = 9, *BC* = 8,*CC* = 7), background 3: n = 29 (*BB* = 9, *BC* = 10,*CC* = 10)). No effects were significant.
(TIF)
