# Supplementary figures and images for: Effects of Cu/Zn Superoxide Dismutase (sod1) Genotype and Genetic Background on Growth, Reproduction and Defense in Biomphalaria glabrata
Source: PLoS Negl Trop Dis. 2012 Jun 19;6(6):e1701. doi: 10.1371/journal.pntd.0001701 (PMC3378597; doi:10.1371/journal.pntd.0001701)

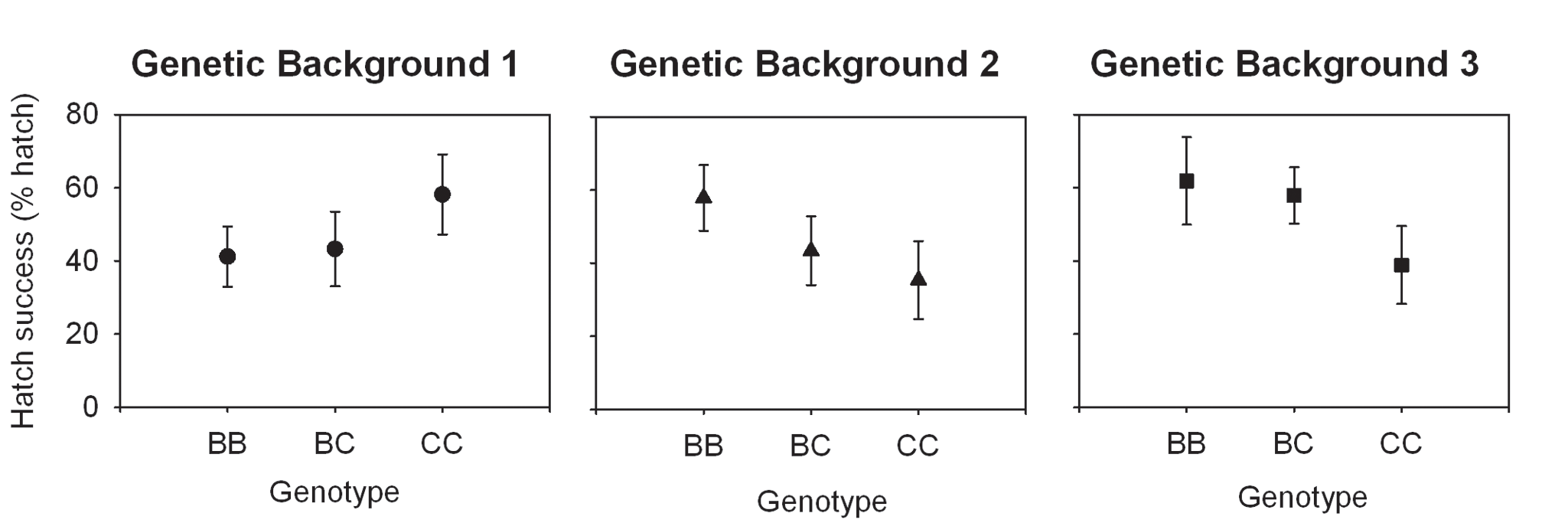

Supplement: Figure S1 — Average hatch success of each genotypic class within each lineage. Means are the average of percent hatch of two clutches per snail across genotypic class, and error bars represent 1±SE (background 1: n = 17 (BB = 8, BC = 6,CC = 3), background 2: n = 24 (BB = 9, BC = 8,CC = 7), background 3: n = 29 (BB = 9, BC = 10,CC = 10)). No effects were significant. (TIF) [file pntd.0001701.s001.tif]
